# Robot-assisted lumbar facet joint infiltration improves accuracy and reduces radiation exposure compared to the manual technique in a comparative phantom study

**DOI:** 10.1038/s41598-026-52435-5

**Published:** 2026-05-12

**Authors:** Michael Kosterhon, Leander Schluechtermann, Florian Ringel

**Affiliations:** 1 Department of Neurosurgery, University Medical Center of the Johannes Gutenberg University, Mainz, Germany; 2 Department of Neurosurgery, LMU University Hospital, Munich, Germany

**Keywords:** Robot-assisted spine intervention, Facet joint infiltration, Medial branch block, Fluoroscopy-guided procedure, Radiation dose reduction, Needle placement accuracy, Spinal injection simulation, Interventional spine robotics, Task Load Index (NASA-TLX), Surgeon experience and learning curve, Chronic pain, Experimental models of disease, Preclinical research, Pain management, Radiography, Chronic pain, Biomedical engineering

## Abstract

Compact robotic systems offer new opportunities for spinal procedures outside the operating room, but their potential for small-scale interventions such as facet joint infiltrations remains underexplored. The objective of this study was to assess the feasibility, accuracy, radiation exposure, and user experience of a compact robotic assistance system (Micromate) for lumbar facet joint infiltrations compared to the manual technique using a standardized phantom model. The robot allows needle placement after a single registration with two orthogonal fluoroscopic images, enabling multiple punctures without repeat imaging. Twenty-two neurosurgeons (0–23 years of experience) performed bilateral infiltrations from L1/2 to L5/S1 both manually and with robotic assistance on an embedded synthetic lumbar spine model. Outcomes included fluoroscopy time, radiation dose, number of X-rays, needle repositioning, and accuracy (Euclidean distance to predefined targets on 3D scans). User experience was evaluated using the NASA-TLX and structured questionnaires. Robotic guidance significantly reduced fluoroscopy time (5.4 vs. 13.7 s), radiation dose (1.95 vs. 3.12 mGy), and X-ray count (6.4 vs. 15.3). Manual infiltrations required multiple needle repositionings (mean 22.2 per participant), whereas there were none in the robotic group (per protocol). Accuracy was higher with robotic assistance (5.9 vs. 7.3 mm deviation; *p* < 0.001), and placement was more consistent across spinal levels. Although total procedure time was longer robotically (27.4 vs. 11.6 min), a learning effect was observed and participants rated their performance higher with the robot. Compact robot-assisted facet joint infiltration is feasible and improves accuracy while reducing radiation exposure. Its potential for multi-level, low-radiation interventions combined with its lower infrastructural requirements may offer a practical and cost-effective alternative to CT-guided procedures, particularly in outpatient settings.

**Supplementary Information:**

The online version contains supplementary material available at 10.1038/s41598-026-52435-5.

## Introduction

The application of robotic systems in spinal surgery has significantly increased in recent years, particularly in the implantation of pedicle screws^1^. The advantages of these technologies lie primarily in enhanced precision, potential reduction of radiation exposure, and reduced intraoperative variability compared to manual procedures^2,3^. However, currently available robotic systems for spinal surgery are often large and primarily designed for use in operating rooms (OR). Most of these systems require pre- or intraoperative CT imaging for navigation and are frequently combined with optical tracking. This necessitates a more complex setup, significant working space, and increased radiation dose, making them less suitable for smaller interventions outside the OR. Consequently, they are rarely used for smaller interventions such as punctures or biopsies. For such applications there is a lack of evidence regarding its feasibility in low-resource, high-volume interventions such as multi-level facet joint infiltrations using only 2D-fluoroscopic registration.

In this study, we investigated the use of a small, flexible robotic system (Micromate, Interventional Systems, Kitzbuehel, Austria) that was designed specifically for interventional procedures outside the OR. While it is normally utilized in conjunction with CT imaging and optical tracking for navigation, a novel feature investigated in this study—which is not yet part of the standard commercial distribution—is the ability to perform needle placement without a preinterventional CT scan. Instead, the robot enables precision-guided needle placement using a straightforward registration process based on only two orthogonal X-ray images. After the initial registration, multiple punctures can be performed on the same patient without the need for repeat imaging, offering potential for radiation dose reduction.

As a model procedure, we selected both manual and robot-assisted facet joint infiltrations (i.e., medial branch blocks). Facet joint infiltration is a well-established minimally invasive technique for both the diagnosis and treatment of facet joint pain. It can also be used to identify patients who may benefit from medial branch radiofrequency ablation^4^. Traditionally, this manual procedure is performed under fluoroscopic guidance to ensure correct needle placement either directly at the joint space or, as in this study, at the medial branch of the posterior spinal nerves. This can result in relevant radiation exposure for both patient and operator, especially when multiple segments are treated or repeated repositioning of needles is required.

The primary objective of this study was to systematically evaluate radiation exposure, intervention duration, and accuracy of both methods. Additionally, subjective parameters such as workload and user acceptance were assessed using the NASA Task Load Index (TLX) and standardized questionnaires.

The results of this study aim to help clarify the potential value of robot-assisted techniques in spinal interventional procedures and to quantify possible advantages in terms of radiation exposure, efficiency, and accuracy.

## Methods

**Fig. 1:**
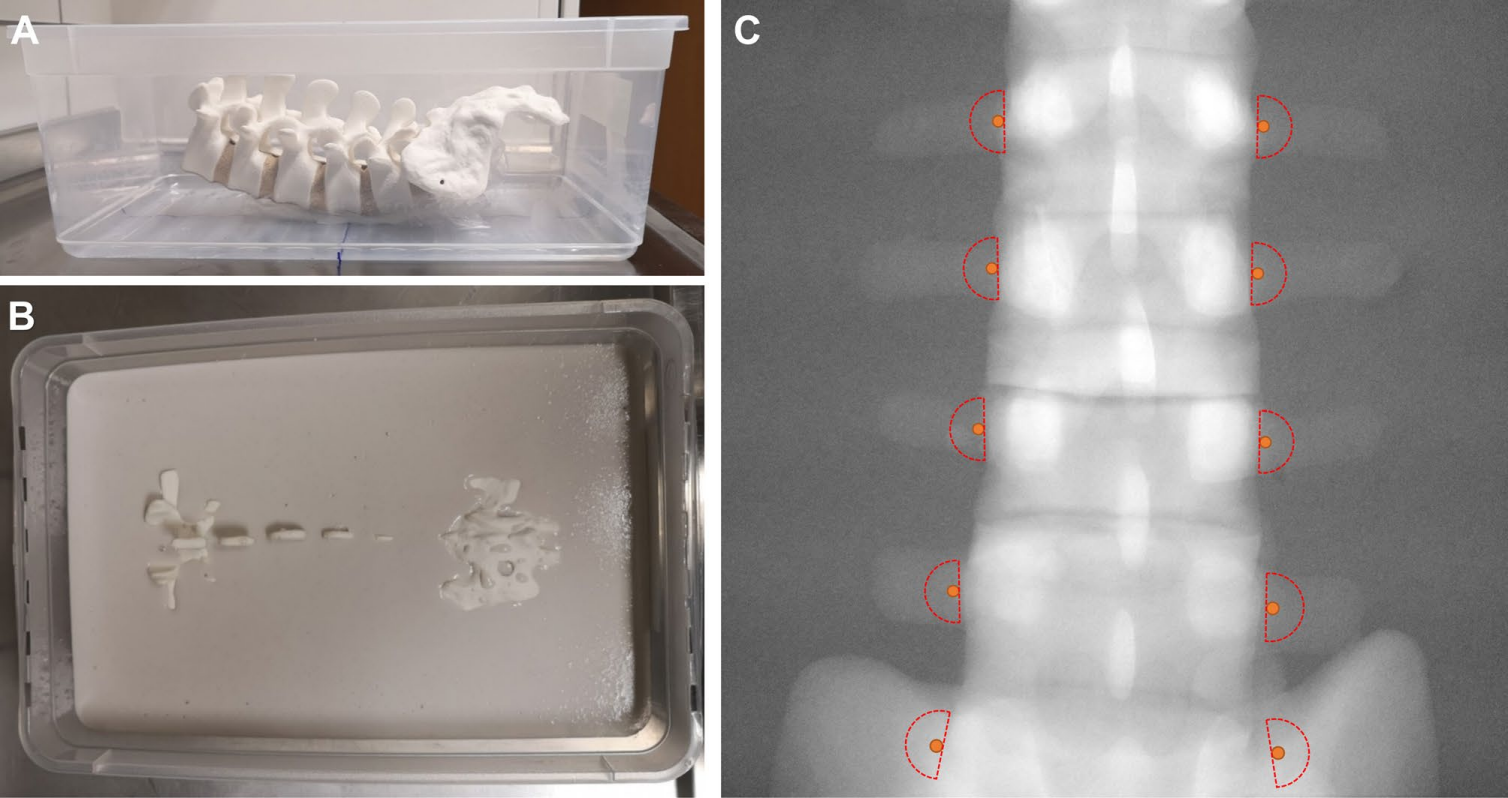
(**A**) Synthetic lumbar spine model embedded in alginate inside a plastic box. (**B**) Cast of the box filled with alginate. (**C**) AP X-ray of the model showing the optimal target points (orange) and the defined tolerance zone (red semicircles), within which the needle tip was accepted during the manual attempt.

A plastic lumbar spine model (SKU 1352, Sawbones, SW. Vashon, Washington, USA) was embedded in alginate (TFC Alginat PREMIUM, white, TFC Troll Factory, Riede, Germany) inside an opaque plastic box (mixing ratio water : alginate = 6 g : 1 g) to simulate a tissue-like consistency (Fig. 1A & B). A total of 22 neurosurgeons with varying levels of professional experience (0–23 years) each performed one manual facet joint infiltration on both sides from L1/2 to L5/S1 following a standardized protocol. The same procedure was then performed using the robotic system.

The infiltration was conducted as a medial branch block. The optimal target point was defined as the junction between the transverse process and the articular process of the facet joint^4,5^, (Fig. 1C). To ensure standardized conditions, all participants were provided with Fig. 1C as a reference template before performing both the manual and the robot-assisted procedures.

### Manual infiltration setup

**Fig. 2:**
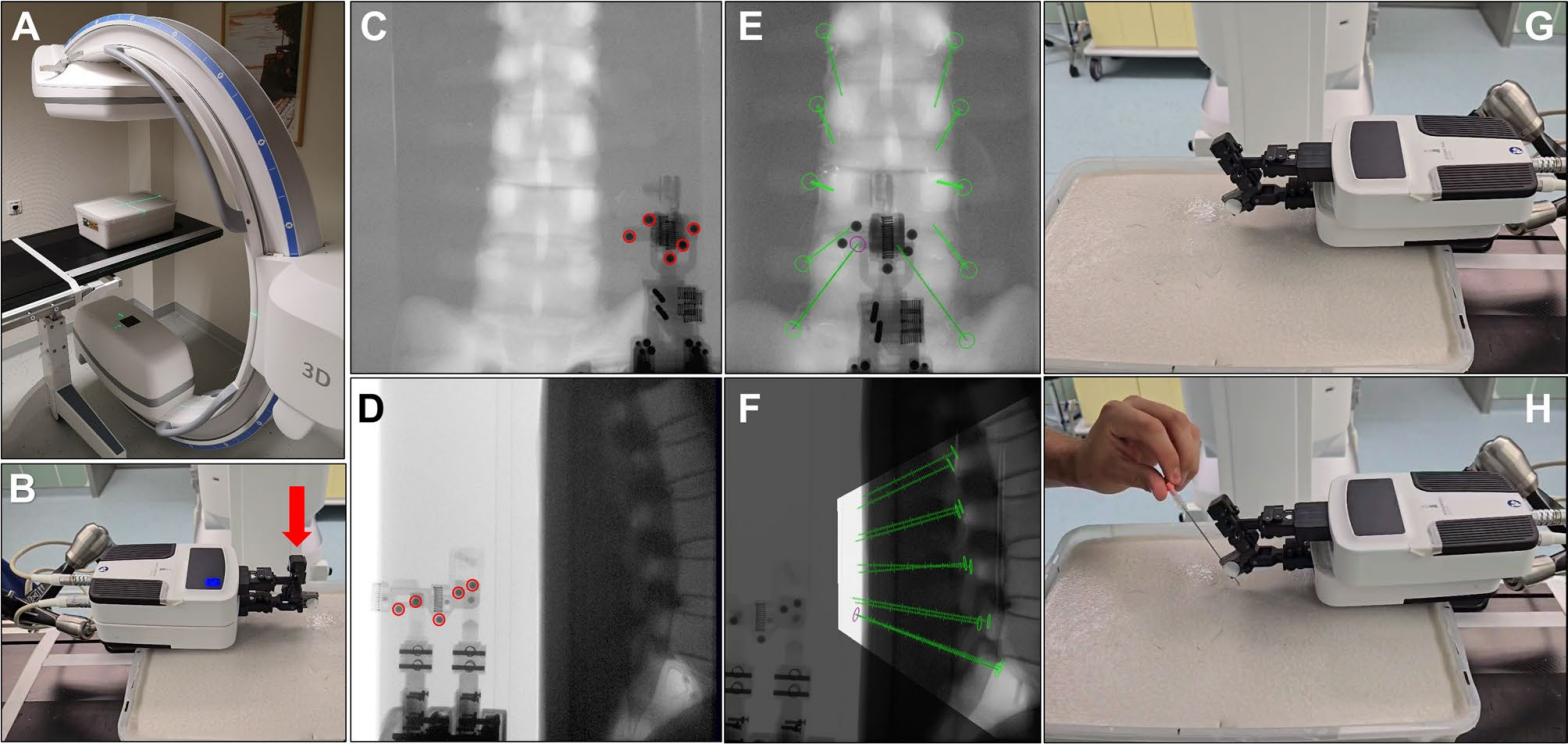
(**A**) Experimental setup with the lumbar spine model placed on a carbon table and the C-arm in AP position. (**B**) Interventional robot mounted above the model using a support arm. Red arrow = movable robotic end-effector. (**C**) AP X-ray image showing the robot’s registration markers. (**D**) Lateral X-ray image showing the robot’s registration markers. (**E**) Planning of needle trajectories in the robot software, AP view. (**F**) Planning of needle trajectories, lateral view. (**G**) Robot aligning itself to the planned trajectory. Note the tilted end-effector. (**H**) Insertion of the puncture needle into the end-effector’s guide.

The phantom model was positioned centrally on a radiolucent table (Fig. 2A). The C-arm (Cios Spin 3D, Siemens, Erlangen, Germany) was set to AP projection. A mark was placed at the level of L4 on the phantom, along with a midline to aid orientation for the participants (analogous to the line connecting the iliac crests when identifying L4 in clinical settings). All participants were provided with a protocol describing how to perform the infiltration. This included a sample X-ray image showing the course of the medial branch and the optimal target point (Fig. 1C). Additionally, a semicircular tolerance zone was defined, within which the needle tip had to appear on the X-ray image to be accepted (Fig. 1C).

Initially, participants prepositioned all needles by inserting them 4 cm vertically into the alginate block, 3 cm to the left and right of the midline at L4. This same procedure was followed for the levels from L1/2 to L5/S1. The vertical spacing between levels was approximately 3 cm. A first X-ray image in AP projection was then taken. Participants were instructed to start at level L1/2 and successively adjust the two needles at each level until bone contact was achieved. A control X-ray image was then taken. If the needle tips projected within the defined tolerance zone (Fig. 1C), participants proceeded to the next level. Otherwise, the needle was repositioned, and a new image was acquired.

The duration of the intervention, the number of needle repositionings, and the number of X-ray images and radiation dose were recorded. Finally, a 3D scan was performed with the C-arm to record the position of the inserted needles in three dimensions. This additional radiation dose was not included in the analysis, as 3D scanning is not performed in clinical practice and was only used here for study purposes.

### Robot-assisted infiltration setup

As none of the participants had used the system before, they received a brief introduction to the robotic system before beginning. A technical assistant was available during the procedure. For the robot-assisted infiltration, the robot was positioned above the model using a mounting arm attached to the radiolucent table (Fig. 2B). The robot consists of a main body and a needle guide (end-effector) that can be adjusted in angle and position. The needle was then inserted manually.

The robot had to be registered in relation to the model. Therefore a software-guided 2D-to-3D workflow was used in which an AP and a lateral X-ray of the phantom were acquired (Fig. 2C & D). The robot was positioned such that its integrated radiopaque markers were clearly visible in both projections (Fig. 2C & D, red circles). By detecting these markers, the software performed a pose estimation to establish the spatial relationship between the robotic end-effector and the C-arm’s coordinate system. Subsequently, all trajectories were defined on the planning workstation by triangulating the target and entry points identified in both orthogonal views (Fig. 2E & F). This method enabled precise 3D-needle guidance based on 2D-fluoroscopy, eliminating the need for volumetric CT data while ensuring a standardized approach across all spinal levels.

Due to technical constraints (see Discussion), the robot was first positioned above the right facet joint line and later, in a second round (requiring repeat registration), above the left facet joint line.

Using the robot’s planning software, all right-sided trajectories from L1/2 to L5/S1 were defined with both target and entry points (Fig. 2E & F). The robot’s end-effector then moved to the first trajectory (Fig. 2G), and the participant manually inserted the needle under slight rotational motion until bone contact was achieved (Fig. 2H). The needle was then clipped just above the alginate surface and the proximal part was removed from the robot to allow movement to the next trajectory. In clinical practice, medication would be injected at this point and the needle removed. However, in this experiment, the needles remained in place for the final 3D scan.

All further trajectories on the right side were approached and punctured in the same manner. The robot was then repositioned over the left side of the model, and the registration, planning, and puncture process was repeated. A final 3D scan was performed after the left-sided infiltrations.

Additionally, both methods were evaluated post-procedure using the NASA-TLX (task load index) via the official online version provided by NASA Ames Research Center^6^. Furthermore a proprietary questionnaire was used for more specific questions. TLX dimensions assessed were: Mental, Physical, and Temporal Demand, as well as Performance, Effort, and Frustration. Participants weighted each dimension, and the weighted TLX total score was calculated. For the questionnaire, questions were structured on a 6-point Likert scale or a 2-point scale when a choice between the two methods was required.

### Evaluation

**Fig. 3:**
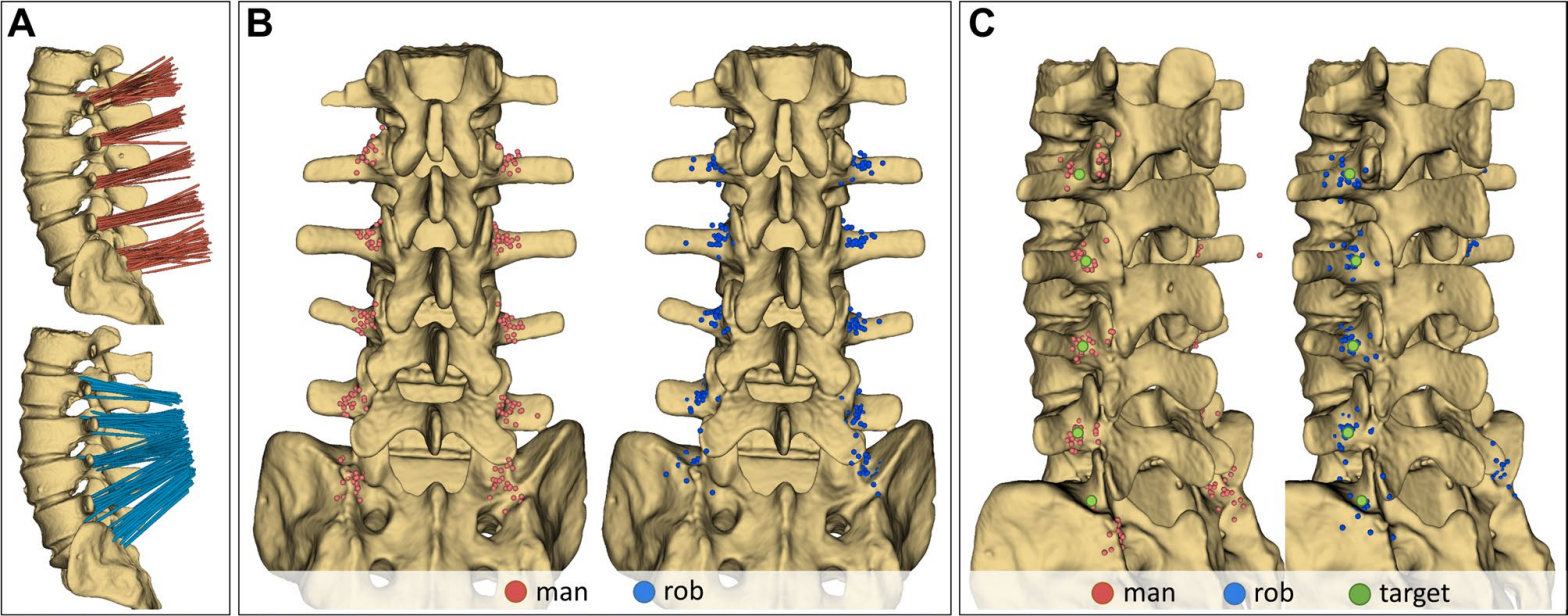
(**A**) Superimposed visualization of all needle placements for the manual method (red) and the robot-assisted method (blue). (**B**) Needle tip coordinates from a dorsal view (red: manual, blue: robot). (**C**) Oblique latero-dorsal view of needle tip coordinates with overlaid ideal target points (red: manual, blue: robot, green: ideal target). Images were generated with the software 3D Slicer (Version 5.8.0, www.slicer.org)^7^.

The 3D scans from both methods were imported into the software 3D Slicer (Version 5.8.0, www.slicer.org)^7^. As each scan showed the same anatomical lumbar spine model, datasets were registered to align the vertebral column. The surface of the spine and needles were segmented using threshold-based methods (Fig. 3A). Marker points were placed at the needle tips, and their 3D coordinates were saved (Fig. 3B). Prior to the experiment, additional marker points had been set to define the ideal target positions for each level and side on the model spine (Fig. 3C). The distance vectors from each needle tip to the corresponding target point were calculated for both methods.

A qualitative evaluation was also performed using three categories: Category 0: Needle tip in the desired target area, Category 1: Needle tip on the facet joint, Category 2: Needle tip outside the two areas above.

### Statistical analysis

Statistical analysis was performed using SPSS (Version 29, IBM, Armonk, NY, USA) and Orange (Version 3.36, www.orangedatamining.com^8^). For outcomes measured at the individual needle level (targeting accuracy), a linear mixed-effects model (LMM) was employed to account for the nested data structure (multiple needle placements per participant). In this model, procedural ‘Mode’ (robotic vs. manual), ‘Spinal Level’, ‘Side’, and ‘Professional Experience’ were defined as fixed effects, while ‘Participant ID’ was included as a random effect (random intercept) to control for inter-individual variability. Interaction terms (e.g., Mode * Level and Mode * Experience) were included to evaluate the consistency of the robotic benefit.

For parameters recorded as a single total value per procedural run (e.g., total radiation dose, fluoroscopy time), paired-samples t-tests were used to account for the dependent nature of the repeated-measures design. For nominal data, the χ^2^ test was applied. Pearson correlation was used for continuous variables and Spearman correlation for categorical variables. Statistical significance was set at *p* < 0.05.

To assess clinical relevance, effect sizes (Cohen’s d) and 95% confidence intervals (CI) were calculated for all primary and secondary endpoints. For the mixed-effects model, d was estimated using the residual standard deviation. Clinical success was further evaluated using Odds Ratios (OR) based on a 10 mm accuracy threshold.

All methods were carried out in accordance with the relevant local and institutional guidelines and regulations. The study was approved by the local ethics committee of Rhineland Palatinate (approval number: 2024–17772). Informed consent was obtained from all participants.

## Results

### Fluoroscopy time and radiation dose

**Fig. 4:**
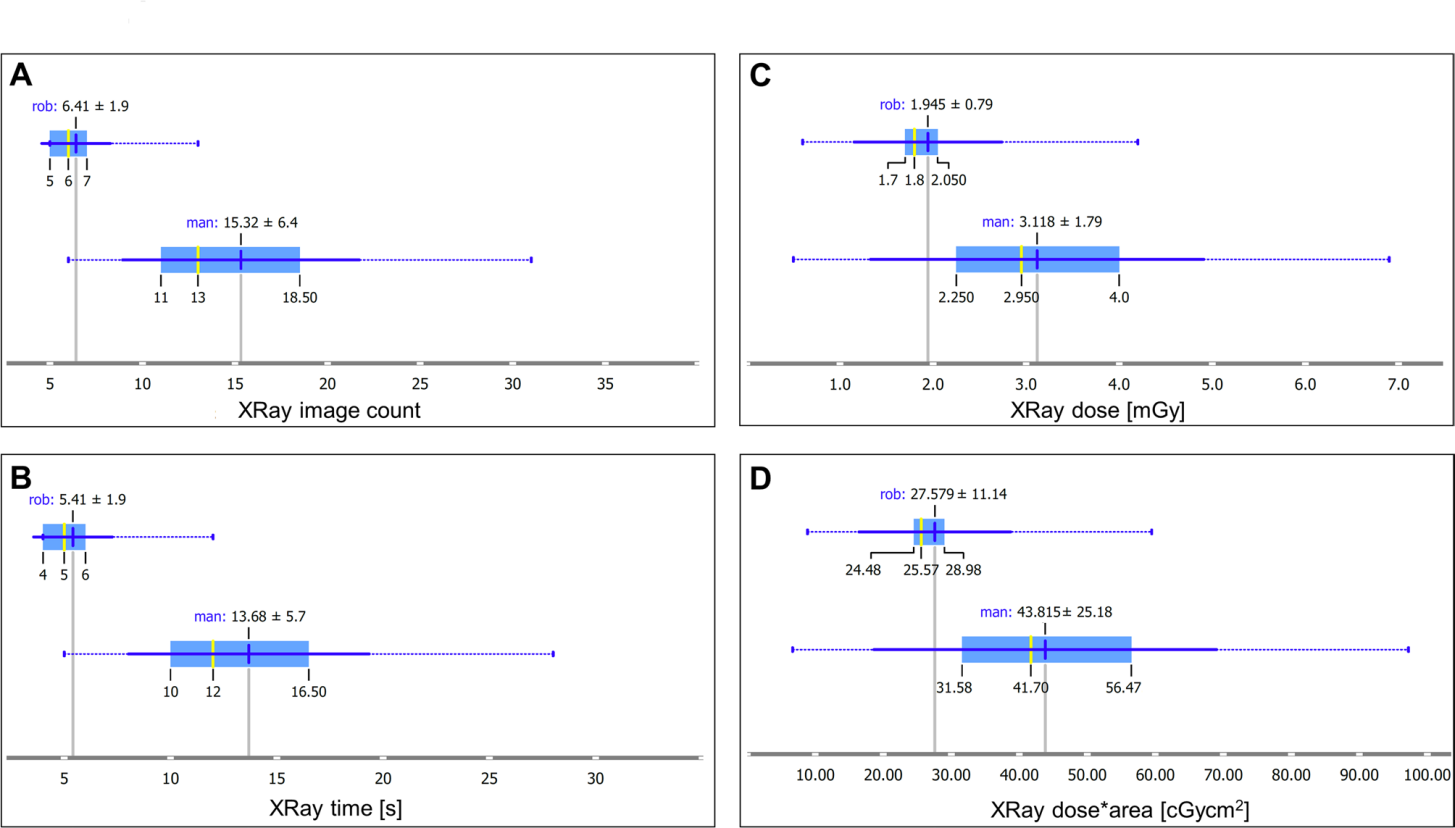
(**A**) Boxplot of the number of X-ray images per method. (**B**) Boxplot of fluoroscopy time. (**C**) Boxplot of radiation dose. (**D**) Boxplot of dose-area product.

In the robot-assisted group, fluoroscopy time was significantly lower compared to the manually guided infiltration (5.4 ± 1.9 vs. 13.7 ± 5.7 s) (*p* < 0.001). On average, 6.4 ± 1.9 X-ray images were acquired during robot-assisted infiltration, compared to 15.3 ± 6.4 during manual infiltration (*p* < 0.001). Consequently, radiation doses were also significantly lower in the robotic group (1.95 ± 0.79 vs. 3.12 ± 1.79 mGy, mean difference 1.17 mGy; 95% CI: 0.37–1.97 mGy; *p* = 0.006; Cohen’s d = 0.65). The dose-area product was 27.6 ± 11.1 vs. 43.8 ± 25.2 cGycm² (*p* = 0.007) (Fig. 4A–D). This corresponds to a reduction in radiation exposure of 37.5%. For more details see Table 1.

**Table 1:** Clinical protocol for manual and robot assisted infiltration with duration of each step of the procedure as well as comparison of Xray usage (mean ± standard deviation (min – max )).

Duration				
Group	Time [min]			
	Manual		Robot	
		Right side	Left side	Both
Setup				
	**3.98** ± 1.67 (2.02–7.97)	5.97 ± 1.83 (3.28–10.17)	5.37 ± 2.47 (3.22–14.50)	**11.34** ± 3.92 (7.20–24.67)
Planning				
	n.a.	5.66 ± 1.79 (2.48–10.50)	3.85 ± 1.10 (2.12–5.95)	**9.51** ± 2.35 (5.07–14.00)
Infiltration				
Level L1/L2	2.36 ± 1.25 (0.62–5.12)	n.a.	n.a.	n.a.
Level L2/L3	1.43 ± 0.88 (0.33–3.83)	n.a.	n.a.	n.a.
Level L3/L4	1.37 ± 0.90 (0.35–3.57)	n.a.	n.a.	n.a.
Level L4/L5	1.23 ± 0.72 (0.10–2.93)	n.a.	n.a.	n.a.
Level L5/S1	1.20 ± 0.56 (0.28–2.50)	n.a.	n.a.	n.a.
Infiltration total	**7.60** ± 2.46 (3.87–12.83)	3.60 ± 1.36 (2.00–7.53)	2.94 ± 0.51 (1.88–3.85)	**6.54** ± 1.69 (4.05–11.28)
Total				
	**11.58** ± 2.90 (5.88–19.50)	15.23 ± 3.43 (7.93–23.03)	12.16 ± 2.99 (8.38–22.75)	**27.39** ± 5.95 (16.32–45.78)

Xray				
Group	Manual	Robot		
Xray time [s]	**13.68** ± 5.66 (5.00–28.00)	**5.41** ± 1.85 (4.00–12.00)		
Xray images	**15.32** ± 6.39 (6.00–31.00)	**6.41** ± 1.85 (5.00–13.00)		
Xray dose [mGy]	**3.12** ± 1.79 (0.50–6.90)	**1.95** ± 0.79 (0.60–4.20)		
Xray dose*area [cGycm²]	**43.82** ± 25.19 (6.67–97.22)	**27.75** ± 11.38 (8.86–59.47)		

### Needle repositioning

In the manual group, an average of 22.2 ± 9.5 needle repositionings per participant were required until all 10 needles were correctly placed. In the robot-assisted group, no repositioning was performed according to the protocol.

### Deviation from the ideal target point

**Fig. 5:**
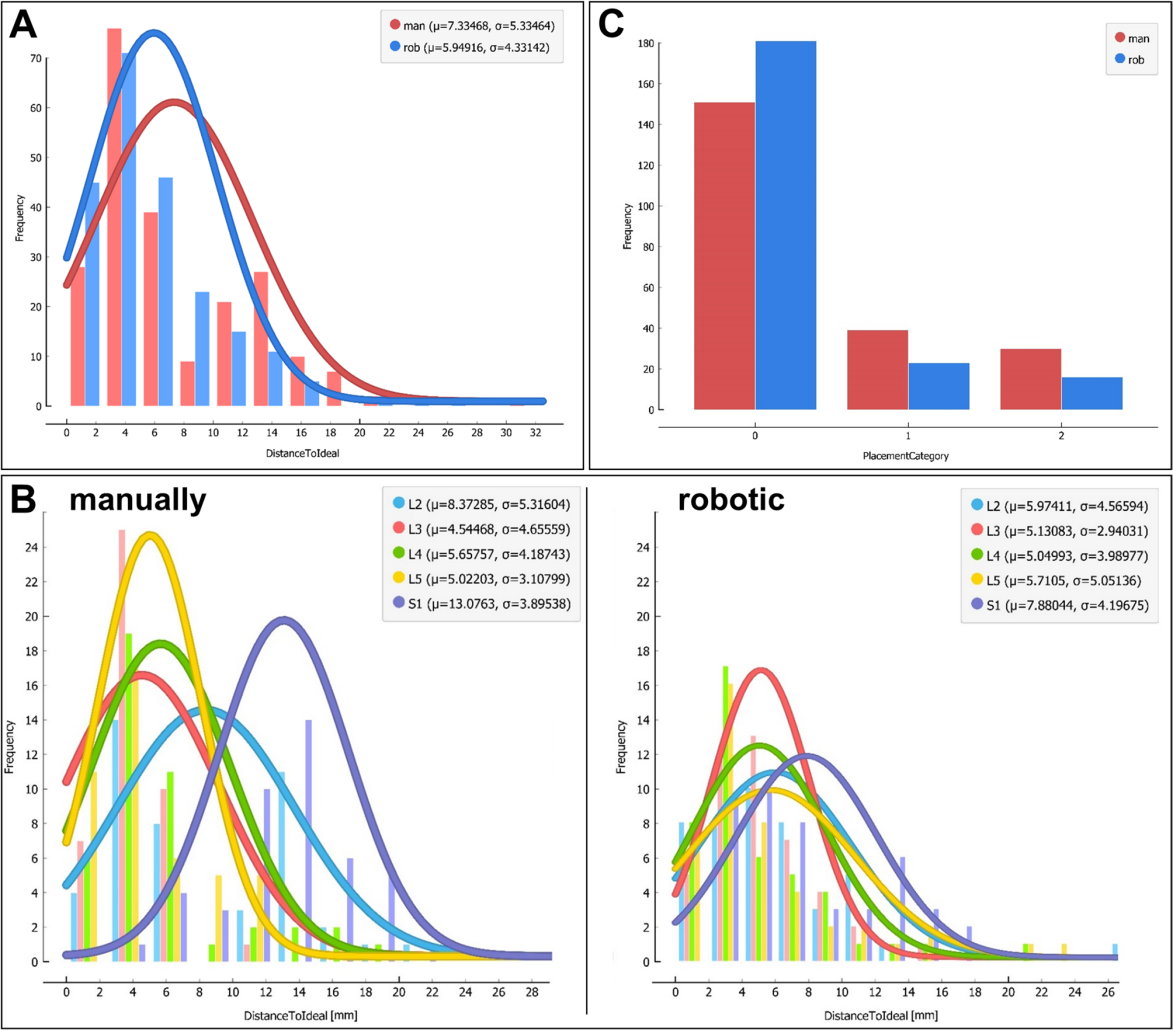
(**A**) Histogram of the Euclidean distance between needle tips and ideal targets with fitted distribution curves (red: manual, blue: robot). (**B**) Histograms of Euclidean distance stratified by spinal level; left: manual, right: robot-assisted. (**C**) Frequency distribution of placement categories for both methods (Category 0: within target area, Category 1: on facet joint, Category 2: outside both areas).

The Euclidean distance (ED) between each needle tip and its respective predefined ideal target point was calculated to assess targeting accuracy. The 3D analysis revealed a smaller distance between needle tips and target points in the robot group: 5.9 mm ± 4.3 (95% CI: 5.38–6.52 mm) vs. 7.3 mm ± 5.3 (95% CI: 5.38–6.52 mm) in the manual group. The linear mixed-effects model revealed that this difference was highly significant (F(1,427) = 14.62, *p* < 0.001, Cohen’s d = 0.32) (Fig. 5A).

Beyond the main effect, the model revealed a significant interaction between procedural mode and spinal level (F(4,427) = 7.26, *p* < 0.001), indicating that the robot-assisted method provided more consistent accuracy across all anatomical heights (Fig. 5B right). In contrast, the manual method showed significantly larger deviations at specific levels, particularly at S1 (anatomically challenging to identify radiographically) and L2 (where mechanical obstruction at the facet joint frequently occurred) (Fig. 5B left, Fig. 3C). Furthermore, a significant interaction between mode and professional experience was observed (F(1,427) = 4.17, *p* = 0.042), suggesting that the precision benefit of robotic assistance was particularly pronounced depending on the surgeon’s expertise.

### Qualitative evaluation

In the robot-assisted group, 181 needles (54.52%) were located within the predefined target area (Placement Category 0), while 151 needles (45.48%) reached this category in the manual group. 23 needles (37.10%) vs. 39 needles (62.90%) were placed directly on the facet joint (Category 1). 16 needles (34.78%) vs. 30 needles (65.22%) were placed outside the defined areas (Category 2). A Pearson χ2 test confirmed a significant difference in the distribution of placement categories between the two procedural modes (χ^2^(2) = 11.08, *p* = 0.004). To address the clinical relevance of these findings, we calculated the Odds Ratio (OR) for achieving a “clinical hit” (Category 0), which was 2.12 (95% CI: 1.35–3.33) in favor of the robotic system. This indicates that the odds of achieving ideal needle placement were more than doubled with robotic assistance. (See Fig. 5C).

### Procedure duration

**Fig. 6:**
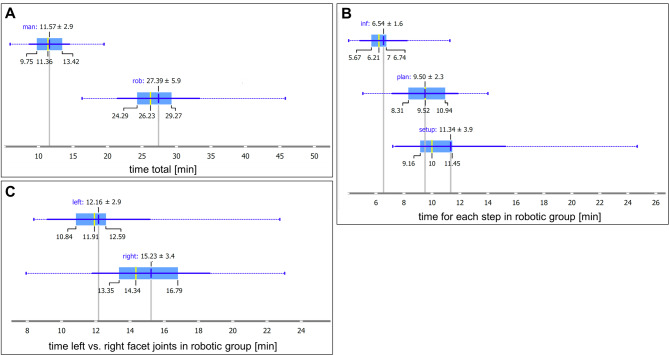
(**A**) Boxplot of total procedure time per method. (**B**) Boxplot of time subdivisions in the robot group: inf = needle insertion, plan = planning phase, setup = robot setup and registration images. (**C**) Boxplot comparing infiltration times for the right (first performed) and left (second performed) facet joint series in the robot group.

The total intervention time was significantly shorter for the manual method, averaging 11.6 ± 2.9 min, compared to 27.4 ± 6.0 min for the robot-assisted method (mean difference 15.8 min; 95% CI: 12.8–18.8 min, *p* < 0.001, Cohen’s d = − 2.33) (Fig. 6A). In the robot group, time was distributed as follows: 11.3 ± 3.9 min for setup, 9.5 ± 2.4 min for planning, and 6.5 ± 1.7 min for actual needle insertion (Fig. 6B). The pure infiltration time was 7.6 ± 2.5 in the manual group and 6.5 ± 1.7 min in the robotic group and did not differ significantly (*p* = 0.128).

The infiltration time for the second (left-sided) procedure in the robotic group was significantly shorter than for the first (right-sided) procedure: 12.2 ± 3.0 min vs. 15.2 ± 3.4 min (*p* < 0.001), indicating a learning effect (Fig. 6C). For more details see Table 1.

### Impact of professional experience

Consistent with the interaction effect observed in the mixed-effects model (*p* = 0.042), further analysis of the manual group revealed that greater professional experience correlated with fewer needle repositionings (Pearson *r* = − 0.43, *p* = 0.048, FDR = 0.254). Correspondingly, fluoroscopy times tended to be lower among more experienced participants (Pearson *r* = − 0.37, *p* = 0.095, FDR = 0.287).

Participants with more than 6 years of experience (*n* = 10, equivalent to board certification in neurosurgery in Germany) achieved Placement Category 0 in 75% of needles in the manual group, compared to only 63.3% among participants with less than 6 years of experience (*n* = 12). This difference was not statistically significant (*p* = 0.056).

With regard to deviation from the ideal target, participants with more than 6 years of experience had a mean distance of 6.7 mm ± 5.1 vs. 7.9 mm ± 5.5 in the less experienced group. This difference was also not significant (*p* = 0.112).

Interestingly, a moderate negative correlation was observed between experience and planning time in the robot group (*r* = -0.60), suggesting that more experienced participants needed more time to become familiar with the robotic system (uncorrected *p* = 0.003, FDR = 0.148).

### Evaluation of questionnaires and TLX

#### NASA task load index (TLX)

The weighted total TLX score revealed no significant differences in perceived workload between the two methods (46.8 ± 15.4 vs. 41.4 ± 15.7) (*p* = 0.257).

However, the TLX dimension Performance (“How successful were you in performing the task? How satisfied were you with your performance?”; 0 = perfect, 100 = failure) was significantly better for the robot group (27.95 ± 18.1) than for the manual group (43.86 ± 24.1) (*p* = 0.018).

All other TLX dimensions showed no statistically significant differences.

In the manual group, the TLX dimension Effort showed a moderate negative correlation with participant experience (*r* = -0.53, uncorrected *p* = 0.0117, FDR = 0.156).

#### Questionnaire

A complete summary of all responses can be found in Supplementary Table 1. Participants were asked how confident they felt after each procedure—manual (Q13) and robot-assisted (Q17). The average score was 2.8 ± 1.3 in the manual group and 2.3 ± 0.7 in the robot group (scale min/max = 1/6). This difference was not statistically significant (*p* = 0.130).

However, the majority of participants (Q23) reported feeling more confident using the robot (72.7%) than with the manual method (27.3%).

Regarding perceived precision during needle placement (Q22), 81.8% voted in favor of the robotic method.

The question of which method participants would prefer to use in the future (Q24) resulted in a tie: 11 for robot-assisted, 11 for manual. When breaking this down by experience (under/over 6 years), the tie remained: 6/6 for < 6 years, 5/5 for ≥ 6 years.

Participants who rated themselves as less confident in Q6 (values between 3 = somewhat confident and 6 = very unconfident) tended to prefer the robotic method: 6 of 9 participants (66.7%) in this group chose the robot.

Conversely, among the 13 participants who rated themselves as very confident (Q6 = 1 or 2), 8 (61.5%) preferred the manual method.

Finally, in alignment with the measured intervention times, 68.2% of participants felt that they were faster using the manual method (Q25).

## Discussion

### Summary of key findings

Robot-assisted infiltration was associated with significantly reduced radiation exposure and improved accuracy in needle placement. A greater proportion of needles were placed within the predefined target zone in the robotic group. However, the total procedure time was significantly longer with robot assistance. Subjective evaluations indicated higher perceived precision and performance with the robotic approach, while overall workload was rated similarly between both methods. A learning effect was observed in the robotic group, with faster execution during the second procedure. Professional experience influenced performance primarily in the manual group, particularly regarding the number of repositionings and radiation exposure.

### Comparison with prior studies

The robotic system presented here has previously been evaluated in phantom models in two published studies^9,10^. However, in those studies, imaging was either performed using CT or navigation was supported by an optical tracking system. Both modalities are often unavailable in outpatient settings or entail increased logistical effort.

What is novel in this study is the registration of the robot using only two fluoroscopic images for performing infiltration procedures across multiple spinal levels. This offers the potential for considerable radiation dose reduction.

### Radiation exposure

It was shown that the robot-assisted method led to a significantly reduced radiation burden. A comparable dose reduction was reported in a study using a positioning device, where the dose was reduced from 2.01 mGy (range 1.61–2.42) to 1.36 mGy (range 0.91–1.58)^11^. However, in that study, only a single level (L4/5 or L5/S1) was infiltrated. Extrapolated to our setting, this would result in an overall dose of ~ 10.05 mGy vs. 6.80 mGy for 5 levels. That study reported a dose reduction of 32.7%, compared to 37.5% in our work.

Another study compared CT-guided and fluoroscopy-guided infiltrations in terms of radiation exposure^12^. In the CT-guided group, the effective dose was 1.59 mSv, whereas in the fluoroscopy group, it was 0.19 mSv. Using a typical lumbar spine conversion factor (k = 0.015–0.020), this corresponds to approximately 80–106 mGy for CT and 10–13 mGy for fluoroscopy. Our study falls well below these levels.

### Procedure time

While total procedure time was significantly longer in the robot-assisted group, it must be noted that all participants were using the system for the first time and thus required more time for planning. Notably, a significant time reduction was already observed in the second (left-sided) robot-assisted procedure, which indicates a learning effect. Since a large part of the total time was dedicated to planning, further time reductions can be expected with repeated use.

This also suggests that robot-assisted infiltration may become especially efficient when treating multiple levels in one session, as planning is required only once and the actual puncture can be performed relatively quickly.

In the aforementioned study by Beyer et al.^9^, the intervention robot was used under CT guidance and compared with freehand CT-guided facet joint injection. The robot-assisted method required 259 ± 111 s (mean: 4.3 min), while the freehand method took 119 ± 77 s (mean: 2.0 min). Up to four corrections were needed per case. These times refer to a single facet injection (only one side). Scaled to our study design with 10 infiltrations, the projected total time would be 43 min (robot) vs. 20 min (manual), and roughly 10 corrections.

### Needle accuracy

Needle positioning accuracy was significantly better in the robot group. Moreover, the results were more homogeneous across all spinal levels than in the manual group.

In the two CT-based studies^9,10^, deviations from the target were reported as: Axial: 0.35 mm ± 1.1; Sagittal: 2.15 mm ± 1.2–2.1 mm ± 0.75 (based on 1 mm CT slice thickness). Unfortunately, Beyer et al. did not report specific numbers for the freehand method but stated that results were significantly worse.

In our study, deviations were 5.9 mm ± 4.3 (robot) and 7.3 mm ± 5.3 (manual), which are clearly higher than those reported above. Unlike studies that use high-resolution CT or 3D-fluoroscopy for registration and planning, our protocol relied exclusively on two orthogonal 2D-fluoroscopic images. This was a deliberate choice to evaluate the system’s potential for reducing radiation and its feasibility in environments where CT is unavailable. The inherent loss of volumetric information in 2D-planning naturally leads to a higher targeting error compared to 3D-CT-based workflows.

However, considering that the target structure comprises an area of approximately 10 mm and that injected medications diffuse locally, we regard these values as clinically acceptable. A standard injection volume of 1–2 ml per facet joint corresponds to a spherical diffusion radius of 12.4 mm–15.6 mm in diameter. Supporting this clinical interpretation, the robotic group demonstrated a significantly higher likelihood of achieving a ‘clinical hit’ (category 0, deviation ≤ 10 mm) with an Odds Ratio of 2.12 (95% CI: 1.35–3.33). This indicates that the odds of ideal needle placement were more than doubled using robotic assistance. While the mean difference was 1.4 mm (Cohen’s d = 0.32), the primary clinical benefit of the robot lies in the standardization of results and the prevention of significant outliers beyond the 15 mm diffusion radius (category 2).

### User experience evaluation

The overall TLX scores showed no significant difference between methods, indicating comparable workload. Among the individual TLX dimensions, only the Performance dimension—assessing satisfaction with one’s own performance—was rated significantly better in the robotic group. Overall, participants felt equally confident using both methods.

Interestingly, less experienced physicians who initially rated themselves as less confident in performing facet infiltrations tended to prefer the robot-assisted method. However, across all participants, preferences for future use were evenly split.

### Target selection

We chose medial branch block over direct facet joint infiltration as the target procedure because the former provides a more clearly defined anatomical landmark.

Clinical studies have shown no significant difference in analgesic efficacy between the two approaches^13^. However, it has been demonstrated that patients who respond positively to a medial branch block also respond better to subsequent radiofrequency ablation of the medial branch than those who initially received facet joint injections^14^. Furthermore, after successful facet infiltration, patients often undergo thermal coagulation of the medial branch, which is the standard target for denervation.

### Methodological rationale

To evaluate the native accuracy of the robotic system, the study protocol did not permit needle repositioning in the robotic group. This constraint was implemented to isolate the system’s intrinsic precision from secondary manual adjustments, providing a clear measure of the ‘single-shot’ success rate. While intra-procedural verification and potential repositioning are standard in clinical practice, as seen in our manual group, the robotic workflow aimed to demonstrate a standardized ‘ideal’ process. This protocol choice allowed for a direct assessment of whether the robotic 2D-to-3D registration alone is sufficient for clinical targets without iterative fluoroscopic feedback. However, we acknowledge that this difference in protocol contributes to the observed reduction in radiation exposure and procedure time per needle in the robotic group.

### Clinical relevance

Compared to manual infiltration, the robot-assisted approach demonstrated reduced radiation exposure, higher placement accuracy, and fewer needle repositionings. These factors contribute to greater procedural efficiency and improved patient comfort by potentially minimizing local tissue trauma. Furthermore, our results suggest a significant potential for procedural standardization: while manual performance was influenced by professional experience—particularly regarding radiation dose and repositioning—robotic assistance provided consistent accuracy across all participants regardless of their seniority. However, the widespread adoption of such technology involves clear economic and logistical considerations. While the system’s compact design and reliance on standard 2D-fluoroscopy reduce the need for expensive infrastructure like CT-suites, clinicians must account for initial acquisition costs and recurring expenses for maintenance and technical support. Additionally, our findings regarding the observed learning effect emphasize the necessity of structured training protocols to ensure smooth integration into clinical workflows.

Given these advantages and despite these implementation requirements, this 2D-fluoroscopy-based method could serve as a lower-radiation, less resource-intensive alternative to CT-guided spinal interventions, particularly for multi-level procedures in outpatient settings where high-end imaging and specialized expertise may be less accessible.

### Limitations

This study has several limitations that must be considered when interpreting the results.

First, the investigation was conducted on a synthetic spine model embedded in alginate, and therefore does not account for potential patient movement during actual clinical procedures. In practice, such movement—due to breathing, discomfort, or involuntary motion—can significantly affect accuracy and registration reliability. For clinical application, patients would need to be appropriately immobilized, for example using flexible restraints.

Second, due to the limited working range of the robotic system used in this study, it was not possible to cover both sides of the spine simultaneously. As a result, the robot had to be re-registered for the left and right facet joint series separately. This added time to the overall procedure and may not reflect future systems with extended working ranges.

Third, some needle deviation was observed during insertion, likely due to the beveled tip of the needles. This was partially compensated for by rotating the needle gently during insertion. However, this kind of manual compensation may not be consistently reproducible in all clinical users, especially in more challenging anatomical regions. Another option might have been to use diamond-tipped needles but unfortunately those were not available in the desired length.

Fourth, while the model allowed for standardization and reproducibility, the alginate material used has a higher radiodensity than real soft tissue, primarily due to its calcium content. This could affect radiation absorption and image contrast, potentially limiting the direct comparability of radiation dose data to clinical practice. Nonetheless, the material was chosen because it offers a favorable contrast with the plastic of the spine model, is cost-effective, and simulates soft tissue consistency reasonably well.

Fifth, during registration, the robot’s markers were not always reliably detected on the fluoroscopic images. This necessitated additional X-rays in some cases. The difficulty may have been due to the different radiographic properties of the phantom compared to human anatomy. Future software improvements, particularly in marker detection algorithms, could enhance the robustness of the registration process.

Sixth, the sequential, non-randomized study design carries a potential risk of a practice effect. However, this risk is considered minimal as the anatomical targets were predefined and clearly marked for both groups (Fig. 1C), eliminating the need for landmark identification. Furthermore, the workflows differ fundamentally—iterative manual needle navigation versus software-based robotic planning—meaning that familiarity with the phantom provided no direct technical advantage for the robotic task. Notably, any potential fatigue from the initial manual run would have disadvantaged the robotic group, yet the robotic results remained superior.

Finally, the robot’s current planning workflow is manual and thus more time-intensive. Automated trajectory preplanning—similar to what is used for pedicle screw placement—could significantly reduce planning time and improve clinical efficiency. Integration of such features would make the robot-assisted workflow more competitive with traditional methods, especially in multi-level interventions.

## Conclusion

The results demonstrate that small interventional robotic systems could be highly promising for procedures performed outside of the operating room, particularly due to reduced radiation exposure and potentially more efficient targeting.

Although the overall procedure duration was longer in the robot-assisted group, further improvements in planning software and increased user training are expected to significantly reduce this time. Robot-assisted techniques may be especially beneficial when multiple spinal levels require infiltration, as a single planning phase enables a rapid execution of the subsequent punctures.

Additionally, the reduced need for needle repositioning in the robot group may increase patient comfort.

In the future, procedures that are currently often performed under CT guidance (e.g., medial branch radiofrequency neurotomy) may potentially be performed with a small robot-assisted system while exposing both patients and physicians to significantly less radiation.

## Supplementary Information

Below is the link to the electronic supplementary material.

Supplementary Material 1

## Data Availability

The datasets generated and/or analyzed during the current study are available from the corresponding author upon reasonable request.
