# Supplementary material for: Robot-assisted lumbar facet joint infiltration improves accuracy and reduces radiation exposure compared to the manual technique in a comparative phantom study
Source: Sci Rep. 2026 May 12;16:14746. doi: 10.1038/s41598-026-52435-5 (PMC13168301; doi:10.1038/s41598-026-52435-5)
Supplement: Supplementary file 1 — Supplementary Material 1 [file 41598_2026_52435_MOESM1_ESM.pdf]

Supplementary Table 1: Results of the questionnaires

No.	Question			
	**Survey before the trial**	**Mean**	**Min / Max**	**SD**
6	How confident do you feel in performing facet joint infiltrations overall?	2.9	1 / 6	1.6
7	How confident do you feel in locating the correct puncture site (entry points)?	2.9	1 / 6	1.5
8	How confident do you feel regarding the correct needle depth?	3	1 / 6	1.5
9	How confident do you feel regarding the correct target point under X-ray?	2.8	1 / 6	1.3
11	Estimated average fluoroscopy time per level (e.g., L4/5 bilaterally) in seconds?	2.6	1.5 / 5	1
12	Estimated number of times a needle needs to be repositioned until correct?	2.8	1 /10	2
10	What type of infiltration do you perform most frequently?	**Facet joint:** 17 (77.3%)	**Ramus medialis:** 3 (13.6%)	**None:** 2 (9.1%)
	**Survey after manual facet joint infiltration**	**Mean**	**Min / Max**	**SD**
13	How confident did you feel in performing facet joint infiltrations overall?	2.8	1 / 5	1.3
14	How confident did you feel in locating the correct puncture site (entry points)?	2.6	1 / 5	1.1
15	How confident did you feel regarding the correct target point under X-ray?	2.5	1 / 5	1.1
16	How confident did you feel regarding the correct needle depth?	2.5	1 / 5	1.2
	**Survey after robot-assisted facet joint infiltration**	**Mean**	**Min / Max**	**SD**
17	How confident did you feel in performing robot-assisted facet joint infiltrations overall?	2.3	1 / 3	0.7
18	How would you rate the overall handling of the Micromate robotic assistance system?	2.4	1 / 4	0.7
19	How would you evaluate the robot setup process?	2.5	1 / 5	0.8
20	How would you evaluate the planning process using the robot?	2.4	1 / 4	0.8
21	How would you evaluate needle insertion using the robot?	2	1 / 3	0.6
	**Comparative questions after the trial**	**Robot**	**Manual**	
22	Which method did you perceive as more precise for needle placement?	18 (81.8%)	4 (18.2%)	
23	Did you feel safer with the robot-assisted procedure compared to the manual one?	16 (72.7%)	6 (27.3%)	
24	Which infiltration method would you prefer to use in the future?	11 (50%)	11 (50%)	
25	With which method did you feel faster overall?	7 (31.8%)	15 (68.2%)	
Note: Questions 1-5 were general questions about professional experience - results in main text				
